# Catalytic High‐Speed Reactive Extrusion for Polyethylene Mechanochemical Upcycling

**DOI:** 10.1002/cssc.70622

**Published:** 2026-04-26

**Authors:** Mansoureh Jamalzadeh, Patrick Masembe, Hrushikesh Pujari, Luca Keller, Miguel Gonzalez Borja, Masud M. Monwar, Lily Cui, Basseem Hallac, Daniel Schwendemann, Margaret J. Sobkowicz

**Affiliations:** ^1^ Plastics Engineering Department University of Massachusetts Lowell Massachusetts; ^2^ Chevron Phillips Chemical Company Kingwood Texas; ^3^ Eastern Switzerland University of Applied Sciences (OST) Rapperswil‐Jona Switzerland

**Keywords:** catalytic reactive extrusion, high shear extrusion processing, polyethylene recycling, specific mechanical energy

## Abstract

This study explores a mechanochemical strategy for polyethylene (PE) recycling using ultrahigh‐speed twin screw extrusion (TSE), focusing on the synergistic effects of high shear rates and catalyst incorporation to induce structural transformations. By systematically controlling specific mechanical energy (SME), a scalable equipment‐independent parameter, the transformation was investigated across two PE structural variations (linear and branched) and linear PE with and without additives. Structural, molecular, and rheological changes were characterized using Fourier transform infrared spectroscopy (FTIR), gel permeation chromatography (GPC), and parallel plate rheology. Catalyst incorporation achieved intimate dispersion and enhanced chain scission, producing low molecular weight liquid and gaseous byproducts collected from the devolatilization zone. Thermogravimetric analysis (TGA) revealed substantial reductions in PE decomposition temperature following catalyst incorporation, attributed to still‐active catalyst sites and the formation of labile functional groups such as ethers and esters. These findings establish TSE as a process intensification approach for controlling PE depolymerization with potential as a preconditioning strategy for downstream chemical recycling. By investing mechanical energy upstream to improve polymer catalyst contact and reduce activation barriers, this approach offers a pathway to lower the thermal energy requirements of chemical recycling, contributing to more economically viable and environmentally sustainable polyolefin waste management at industrial scales.

## Graphical abstract

Twin screw extrusion at high speeds is a mechanochemical strategy for polyethylene recycling that takes advantage of high shear rates and intimate mixing with catalyst to induce structural transformations. This process intensification approach can lower the thermal energy requirements of chemical recycling, contributing to more economically viable and environmentally sustainable polyolefin waste management at industrial scales.

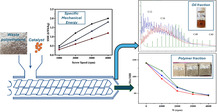

## 1 Introduction

Polyolefins, the largest class of commodity plastics, are valued for their versatility and cost‐effectiveness. However, improper waste management practices can lead to plastic waste accumulation and pose serious environmental challenges [1]. Addressing this issue requires comprehensive plastic waste management strategies, including reuse, repurposing, recycling, upcycling, and energy recovery through incineration as a last resort [1]. The feasibility of these strategies varies depending on the application; for instance, thin films present particular challenges for reuse due to their limited durability and functional constraints, making recycling or alternative end‐of‐life treatments more practical options [2].

While the core technologies are not new, polyolefin recycling has garnered recent research attention, with efforts focusing on both mechanical and chemical recycling approaches. Mechanical recycling is generally considered more economically viable and less energy intensive than chemical recycling [3]. The process involves collection, sorting, washing, downsizing, and extrusion, followed by subsequent product forming operations [4]. However, mechanical recycling of polyolefins faces several technical limitations due to both polymer degradation during processing and the heterogeneous nature of postconsumer waste streams [5, 6]. Even with clean, single‐polymer inputs, recyclability is strongly influenced by the polymer's chemical structure and the presence of additives [7, 8]. While additives are originally incorporated to enhance polymer performance, the chemical complexity arising from different additive formulations across manufacturers can inadvertently promote thermal degradation and complicate end‐of‐life material behavior [6, 9]. In the case of polyethylene (PE) recycling, degradation during extrusion is governed by the interplay between chain scission and branching reactions, which are highly sensitive to processing conditions. Extrusion parameters such as temperature and shear rate can influence the polymer's chemical structure during reprocessing [10, 11]. High thermal and shear energy can induce oxidative formation of free radicals, which react with the carbon backbone to generate macroradicals. These reactive species undergo β‐scission, intramolecular rearrangement, and recombination reactions, leading to molecular weight reduction, branched chain and double bond formation, and, in some cases, crosslinked networks [12, 13, 14]. Furthermore, in oxygen‐rich environments, oxidative degradation introduces polar functional groups such as carbonyls, hydroxyls, and carboxylic acids into the polymer structure, accelerating material aging and embrittlement [15]. Under oxygen‐limited conditions, the degradation pathway shifts toward shear‐induced scission and unsaturation formation, with relatively less branching [16].

The cumulative nature of these degradation pathways, along with the compositional complexity, means that each mechanical recycling cycle progressively narrows the range of viable applications for recycled polyolefins, ultimately confining them to lower‐grade products, a process known as downcycling. Once molecular weight has decreased sufficiently or undesirable structural modifications have accumulated, the material can no longer meet performance requirements for its original application or equivalent uses [17]. Chemical recycling offers an alternative approach that sidesteps these limitations by deconstructing waste polymers into molecular building blocks (monomers, oligomers, or chemical intermediates) that can be purified and repolymerized into virgin‐quality materials or converted into higher‐value products [18, 19]. Among the various chemical recycling technologies, pyrolysis has emerged as the most widely studied approach for polyolefin waste valorization. This process involves the thermal decomposition of polymers in an oxygen‐free environment at temperatures typically ranging from 400°C to 600°C, breaking down long polymer chains into smaller hydrocarbon fractions, including gases, liquids (pyrolysis oils), and solid residues (char) [20, 21]. While pyrolysis theoretically enables upcycling by converting degraded polyolefin waste into valuable chemical feedstocks, the process is inherently energy intensive, requiring high temperatures to overcome the strong carbon–carbon bonds in the polymer backbone.

To reduce energy requirements and improve product selectivity of conventional thermal pyrolysis, catalytic pyrolysis has gained considerable attention as a more efficient alternative. By incorporating catalysts into the pyrolysis process, reaction temperatures can be reduced by 50°C to 150°C compared to noncatalytic systems, while simultaneously enhancing the yield and quality of desired products [22, 23, 24]. Catalyst acidity, porosity, and pore architecture dictate polymer chain accessibility to active sites and enable selective conversion toward specific hydrocarbon fractions such as naphtha‐range hydrocarbons, aromatics, or olefins, depending on the catalyst type and operating conditions [25, 26, 27]. This selectivity is particularly valuable for recycling and upcycling applications, as it allows tailoring of pyrolysis outputs to meet the specifications of high‐value chemical feedstocks rather than producing broad, mixed hydrocarbon streams that require extensive downstream processing. However, challenges such as catalyst deactivation and energy requirements of regeneration must be considered. Even with the use of catalysts, there remains a critical need to further reduce the energy associated with the pyrolysis reaction.

A key yet often overlooked factor affecting catalytic pyrolysis efficiency is the degree of catalyst dispersion within the polymer matrix. The high viscosity, long chains, and poor thermal conductivity of polyolefins inherently limit effective heat and mass transfer, restricting polymer‐catalyst interactions even under optimal conditions [28]. Conventional approaches, such as dry blending catalyst powder with polymer feedstock prior to reactor feeding, often result in heterogeneous catalyst distribution and incomplete polymer catalyst contact [29]. While reactor designs employing fluidization can improve mixing and heat transfer during pyrolysis by suspending particles in a gas stream, these systems still face challenges related to particle size requirements, operational complexity, and energy costs associated with maintaining fluidization conditions [30, 31]. Addressing these mixing limitations upstream of the pyrolysis reactor, rather than relying solely on in‐reactor processes, presents an alternative approach to enhancing polymer catalyst interactions and overall process efficiency.

Recent studies have explored mechanical approaches to modify polymer properties prior to chemical depolymerization [32]. Extrusion pretreatment at elevated temperatures has been shown to alter polymer thermal decomposition dynamics and reduce apparent activation energies for pyrolysis reactions, with studies demonstrating that processing in twin screw extruders modifies molecular weight, melt properties, and subsequent pyrolysis kinetics [33, 34]. However, these studies have primarily focused on polymer degradation and property modification during extrusion, with limited attention to how mechanical processing might address the fundamental catalyst dispersion challenges that limit pyrolysis efficiency. Moreover, a critical gap remains in understanding the energy trade‐offs: while mechanical preconditioning consumes energy through shear and mixing, it may reduce the far greater thermal energy demands of pyrolysis by improving polymer–catalyst contact and lowering activation barriers. Systematic evaluation of these trade‐offs requires quantitative tools capable of relating mechanical processing intensity to downstream pyrolysis performance.

To quantitatively assess these energy trade‐offs and enable process optimization, specific mechanical energy (SME), defined as the net mechanical energy input per unit mass of material processed, provides a robust scaling parameter for characterizing the energy intensity across different processing conditions and equipment configurations. The importance of SME in understanding polymer degradation mechanisms has been demonstrated in studies of ultrahigh‐speed twin‐screw extrusion systems, where researchers isolated the effect of temperature from mechanical shearing by analyzing viscous dissipation energy and local shear rates along the extruder barrel [14]. This study revealed that the extent of molecular weight reduction is governed by the cumulative mechanical energy input, with degradation kinetics strongly dependent on screw speed, screw profile geometry, and residence time. Such mechanochemical effects, traditionally viewed as undesirable degradation in conventional polymer processing, can be strategically harnessed for chemical recycling and upcycling applications where controlled chain scission and enhanced reactivity are beneficial rather than detrimental.

Building on this foundation, the mechanochemical approach represents a paradigm shift for polyolefin recycling by deliberately coupling controlled mechanical energy input with chemical transformation pathways. In this context, the present work investigates the integration of extrusion processing with catalysis within a reactive extrusion (REX) setup to enable polymer degradation in a continuous and solvent‐free process. REX, which employs an extruder as a continuous chemical reactor, has evolved significantly over the past three decades and is now widely used for the chemical modification of polymers [35, 36]. While a twin‐screw extruder traditionally provides conveying, melting, and mixing, REX additionally offers favorable reaction conditions and is well‐suited for processing high‐viscosity polymer melts. The continuous deformation and surface renewal inside the extruder promote efficient mixing and effective heat transfer, enabling shorter reaction times than in stirred vessels. Moreover, the high shear environment inherent to twin screw extrusion can simultaneously achieve intimate catalyst dispersion and initiate polymer chain scission, addressing both the mass transfer limitations and activation energy barriers that constrain conventional pyrolysis efficiency. However, realizing these potential benefits depends on the optimization of key extrusion parameters, including temperature, screw configuration, screw speed, and feed rate, which govern the degradation reaction mechanisms and determine the extent of molecular weight reduction and catalyst distribution.

This study establishes SME as a quantitative framework to control the mechanochemical degradation process by directly relating mechanical energy input to chemical transformation. In this work, we posit that integrating high‐shear mechanical processing with catalytic reactions can increase the interaction between polymer and catalyst through uniform dispersion of the catalyst and increase the temperature in the melt, thereby promoting reaction efficiency. By systematically varying SME through controlled extrusion parameters and correlating it with reaction extent and catalyst dispersion quality, this work aims to determine the effect of mechanical energy to promote depolymerization reactions in preparation for downstream chemical recycling processes. Furthermore, this work investigates how polymer architecture and formulation affect mechanochemical degradation pathways, examining two polyethylene structural variations (branched and linear) and the influence of additives in linear PE to establish generalized relationships between SME, polymer structure, and degradation mechanisms.

While mechanical processing has been shown to induce chain scission and other chemical reactions in polyethylene before, this work uniquely offers the use of ultrahigh shear using a unique twin screw extruder that can rotate up to 4500 rpm. It also examines systematically the combined effects of time, temperature, and shear stress via tracking and variation of SME. Extrusion with and without catalyst and comparing reactions in PE with and without oxidative stabilizers outlines the range of responses to mechanochemical processing. The significance of this work extends beyond process optimization to address fundamental questions about energy efficiency in polyolefin chemical recycling. Ultimately, this research establishes SME as a scalable, equipment‐independent parameter for controlled depolymerization and demonstrates a mechanochemical approach for waste transformation. It offers a potential pathway to reduce the substantial thermal energy burden of chemical recycling by investing mechanical energy upstream, potentially improving both the economic viability and environmental sustainability of polyolefin upcycling at industrial scales.

## 2 Experimental Approach

### 2.1 Material

Three polyethylene grades were provided by Chevron Phillips Chemical for this study. High‐density polyethylene (linear structure) was investigated in two formulations: with commercial additives (HDPE) and without additives (HDPE‐NA). Branched polyethylene was supplied as linear low‐density polyethylene (LLDPE) containing commercial additives. The weight‐average molar mass (M_w_), number‐average molar mass (M_*n*_), and polydispersity index (PDI) of HDPE, HDPE‐NA, and LLDPE are presented in Table 1. The catalyst was a commercially used fluid catalytic cracking (FCC) catalyst, also known as equilibrium catalyst (E‐Cat), which is a byproduct from the FCC process in oil refineries. It is a spray‐dried composite comprising a matrix, binder, and Y zeolite with a BET surface area of 155 m^2^/g, pore volume of 0.4 cm^3^/g, and average particle size of 72 μm.

**TABLE 1:** Molar mass details of three polyethylene grades.

	M_w_, kg/mol	M_*n*_, kg/mol	PDI
HDPE	152	19.0	8.0
HDPE‐NA	141	19.5	7.2
LLDPE	127	48.9	2.6

### 2.2 Processing

Processing was conducted on a co‐rotating, intermeshing twin‐screw extruder (KZW 15 TW, TECHNOVEL Corporation, Osaka, Japan) with a screw diameter of 15 mm, L/D of 60:1, and a maximum speed of 4500 rpm. The eight‐barrel system was equipped with a strand die, water bath, and pelletizer. Experiments were conducted at screw speeds of 1000, 2000, 3000, and 4000 rpm under two flat temperature profiles: 210°C (reference) and 300°C (high‐temperature set). Zone 7 was maintained under vacuum (70 mmHg) for devolatilization, with an ice‐cooled trap to collect condensate. Zone 7 was maintained under vacuum (70 mmHg) for devolatilization, with an ice‐cooled trap to collect condensate.

The maximum feed rate was determined empirically at 1000 rpm by gradually increasing the feed up to the safe torque limit, defined as 80% of the extruder's maximum current draw (85 A). Residence time for each feed condition across all screw speeds was measured by introducing colored tracer pellets at the feed zone and recording the elapsed time until the tracer first appeared at the die exit. Reported residence times represent the average values across the evaluated screw‐speed range. Table 2 illustrates the expected trends: the feed rate increased with temperature due to reduced polymer viscosity, while residence time decreased with the screw speed as a result of decreased screw channel filling. Overall, increasing the feed rate led to shorter residence times.

**TABLE 2:** Feeding rate, maximum safe torque, (F_max_) and 50% of F_max_ for each material and processing temperature, with their corresponding mean residence times measured at 1000, 2000, 3000, and 4000 rpm.

Material	Feed condition	Feed rate, g/min	Average residence Time, s
HDPE	F_max_@210°C	19	75
	F_max_@300°C	30	50
	50F_max_@300°C	15	92
HDPE‐NA	F_max_@210°C	24	49
	F_max_@300°C	40	34
	50F_max_@300°C	20	63
LLDPE	F_max_@210°C	17	73
	F_max_@300°C	30	45
	50F_max_@300°C	15	85

The TSE was programmed with two‐flighted screw elements at various pitches and lengths, as well as different kneading blocks (the screw profile is presented in the supporting information Table S1). In addition, a C.W. Brabender batch mixer (Intelli‐Torque Plasti‐Corder Type: 01‐55−000) was used to assess the impact of temperature and time on oxidative changes to the polymers in the absence of aggressive shear. Polyethylene pellets weighing 40 g were filled into the Brabender and processed at two temperatures (210°C and 300°C) while maintaining a rotor speed of 50 rpm to minimize mechanical shear stress. Samples were collected after 10 and 30 min.

The catalytic reactive extrusion processing was conducted by adding 10 wt% of E‐Cat to HDPE and LLDPE. The processing parameters, outlined in Table 3, were selected based on findings yielding the lowest molecular weight in TSE process. To examine the influence of residence time on catalytic degradation, two distinct feeding rates were applied during reactive extrusion. This allowed for a comparative assessment of how residence time affects polyethylene mass conversion by adding catalysts and shear‐driven conditions. In this step, the HDPE‐NA grade was excluded because commercial polyethylene grades usually contain stabilizing additives. Therefore, the research focused solely on the additive‐containing HDPE, which is more relevant for practical applications.

**TABLE 3:** Processing parameters of catalytic reactive extrusion.

Material	Temperature, °C	Screw speed, rpm	Feeding rate, g/min	Catalyst, wt%
HDPE LLDPE	300	4000	F_max_ 50F_max_	10

### 2.3 Specific Mechanical Energy

In the context of high‐speed twin‐screw extrusion, SME serves as an indicator of the energy transferred into the polymer melt through shear and viscous dissipation. SME is calculated using Equation (1):

(1) $$\mathrm{SME}=\frac{P\times T\times N}{T_{\max}\times N_{\max}\times Q}$$

where *T* is the actual torque applied in amperes (A), *P* is the motor power rating of the extruder in watts (W), *N* is the actual screw speed in rpm, and *Q* is the mass feed rate (kg/h). *T*_max_ and *N*_max_ represent the maximum torque and speed of the twin‐screw extruder, in this case equal to 85 A and 4500 rpm, respectively. The power P was determined by measuring the three‐phase current (I) of the extruder drive motor using a series ammeter under a 180‐volt supply. Furthermore, the power required for an empty TSE at a screw speed ranging from 1000 to 4000 was measured to be between 1.1 and 3.1 kW. This approach enables precise control of SME by adjusting screw speed and feed rate while maintaining a constant barrel temperature setting (though actual temperature may vary due to shear heating). The measurement of current draw by the machine takes into account the torque required to turn the screw and the electrical power supplied to the heaters to heat the barrel. So, the experimental design systematically varied screw speed and feed rate to correlate the applied mechanothermal energy with the chemical structure evolution of polyethylene.

### 2.4 Characterization Methods

#### 2.4.1 Parallel Plate Rheology

The rheological measurements were performed in oscillatory mode using ARES‐G2 rotational rheometer (TA Instruments), fitted with 25 mm stainless steel parallel plates. Samples for the measurements (25 mm diameter disk with a thickness of 1.5 mm) were prepared using either a micro‐injection molding machine (Xplore Instruments BV) for samples from the TSE process (without catalyst) or compression molding for samples from the catalytic reactive extrusion. The samples were preheated for 3 min at a temperature of 200°C before being injected into the mold at a pressure of 6 MPa. While samples from the catalytic reactive extrusion were kept under a pressure of less than 1000 psi for 5 min at a temperature of 200°C before cooling down. Frequency sweep tests were conducted at 200°C with a strain amplitude of 10% and angular frequencies ranging from 0.1 to 100 rad/s under a nitrogen environment. The complex viscosity (η∗) curves were examined to compare the viscoelasticity of polyethylene under different processing conditions.

#### 2.4.2 Size Exclusion Chromatography (SEC)

Molar mass distributions of polyethylene were determined using a PL 220 GPC/SEC high‐temperature chromatography unit (Polymer Laboratories, now an Agilent Company). The unit was operated at a temperature of 145°C with a flow rate of 1.0 mL/minute for 1,2,4‐trichlorobenzene (TCB) as the solvent. BHT (2,6‐di‐tert‐butyl‐4‐methylphenol) was used as a stabilizer in the TCB at a concentration of 0.5 g/L. An injection volume of 400 µL was used with a nominal polymer concentration of 0.5 mg/mL. The sample was dissolved in stabilized TCB by heating it at 150°C for approximately 4 h, with occasional gentle agitation, depending on the dissolution temperature and polymer solution. Three Waters Styrogel HMW‐6E columns were calibrated using the integral method with a broad linear polyethylene standard (Chevron Phillips Chemical Company LP's Marlex RTM BHB 5003 polyethylene) for which the molecular weight distribution had been determined. The concentration was detected using an IR4 detector (Polymer Char, Spain).

#### 2.4.3 High‐Temperature Gas Chromatography (HTGC)

Accumulated devolatilization residues generated during catalytic reactive extrusion were collected at the end of the process for HDPE and LLDPE. These included oily condensates recovered from the vacuum trap and waxy solids deposited in the vacuum ducts and trap, which were analyzed using HTGC.

An Agilent 7890A gas chromatograph (GC) (Agilent Technologies, Santa Clara, CA, USA) with an Agilent 7693A autosampler, cool on‐column (COC) inlet, and flame ionization detector (FID) was used to characterize the carbon number distribution of multiple samples. Wax and oil samples were diluted with carbon disulfide to dissolve as much material as possible and shaken for 60 s, followed by centrifugation for 10 min at 3000 rpm (1750 x g). The liquid portion of each sample was pipetted into a GC vial, and 0.1 µl was injected onto the chromatographic column. The separation was carried out using an MXT‐1HT Sim Dist capillary column (5 m x 0.53 mmID x 0.20 µm df; Cat# 70 115; Restek, Bellefonte, PA). Helium was used as a carrier gas at a constant flow of 18 ml/min. The COC inlet temperature was set to “Track Oven” mode. The oven temperature program was set up as follows: Initial temperature was held at 35°C for 0 min, then increased at 20°C/min to 420°C and held for 10 min. Temperature of the FID was set to 420°C. Data acquisition and processing were done using OpenLab Chemstation (Agilent Technologies) software.

Although a complete mass balance was not achieved for each run, establishing a comprehensive mass balance remains an important objective for future work.

#### 2.4.4 Carbon‐13 Nuclear Magnetic Resonance Spectroscopy (^13^C NMR)

For ^13^C NMR analysis, all the samples were prepared in a 10 mm NMR tube. About 0.25 g of polyethylene (PE) sample was dissolved in a mixture of 2.5 mL of 1,2,4‐trichlorobenzene (TCB) and 0.70 g of 1,4‐dichlorobenzene‐*d*_4_ (DCB‐*d*_4_) for ^13^C NMR data collection. The sample and the solvent mixture were then heated in a heating block at 130°C for 4–5 h. The mixture was occasionally stirred with a stainless‐steel stirrer to ensure homogeneous mixing. The resulting solution was then left overnight (for 15–16 h) in the heating block at 112°C to ensure complete dissolution of the polymer chains. The final concentrations of the resulting solutions were about 5–6 wt%. The samples containing catalyst particles were first dissolved in 5.0 mL of 1,2,4‐trichlorobenzene (TCB) at 130°C. The samples were then left overnight at 115°C for the precipitation of the catalyst particles at the bottom of the tube. About 3.0 mL of the clear solution of each sample was then transferred to a 10 mm NMR tube, and 0.70 g of DCB‐d4 was added to the solution. The mixtures were mixed well at 110°C to prepare a homogeneous solution.

The ^13^C NMR data were collected in a Bruker 500 MHz NMR instrument equipped with a 10 mm cryoprobe fitted with z‐gradient. All the NMR data were collected at 125°C, and the sample was equilibrated at 125°C for 15 min before the start of data acquisition. The data were collected and processed with Bruker's Topspin software (v. 4.1.4). The ^13^C NMR spectra of the polyethylene samples were collected with standard pulse program (*zgig*) from Bruker's pulse sequence library using the standard parameter set, including: a 12.0 µsec 90° pulse width, an 8.2 kHz spectral window, an 8.0 sec relaxation delay, and a 2.0 sec acquisition time. Sufficient transients (1–4 k) were collected to achieve a reasonable signal‐to‐noise ratio. The data was zero‐filled to 131 k data points and exponentially weighted with a 1.0 Hz line‐broadening before Fourier transformation. The data were referenced to PE backbone peak at δ ∼30.0 ppm.

#### 2.4.5 Fourier Transform Infrared Spectroscopy

The chemical structure and the formation of new functional groups were examined using a Nicolet iS50 (Thermo Scientific) FTIR spectrometer in attenuated total reflectance mode. Samples of extrudate were placed under the crystal, and 64 scans were averaged to obtain FTIR spectra. The carbonyl index (CI) was determined for the processed samples by calculating the ratio of the area under the peak at approximately 1740 cm^−1^ to the area under the C–H stretch peak at 2930 cm^−1^.

#### 2.4.6 Thermal Gravimetric Analysis

A Thermogravimetric Analyzer (Mettler Toledo, TGA2‐SF) was used to investigate the decomposition temperature of samples. The measurements were conducted in a nitrogen atmosphere. Samples (6–10 mg) were heated at a rate of 35°C/min under flowing nitrogen from 40 to 600°C.

## 3 Results and Discussion

### 3.1 Specific Mechanical Energy (SME)

**FIGURE 1:**
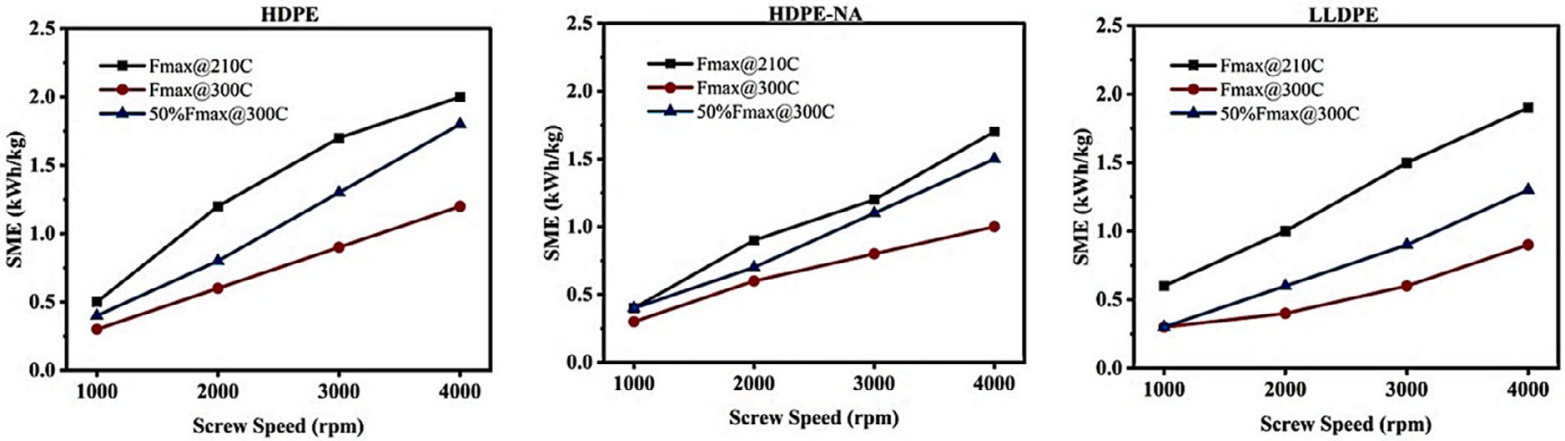
Specific mechanical energy versus screw speed at different feeding rates and temperatures for each polyethylene grade.

The calculated SME for three polyethylene grades subjected to different processing conditions is shown in Figure 1. The SME increases roughly proportionally with screw speed but varies slightly for each resin, with the highest values for HDPE with additives. In general, elevated feeding rates and processing temperatures lead to a reduction in SME due to the shorter residence time and lower viscosity of the melt, respectively.

### 3.2 Rheology

**FIGURE 2:**
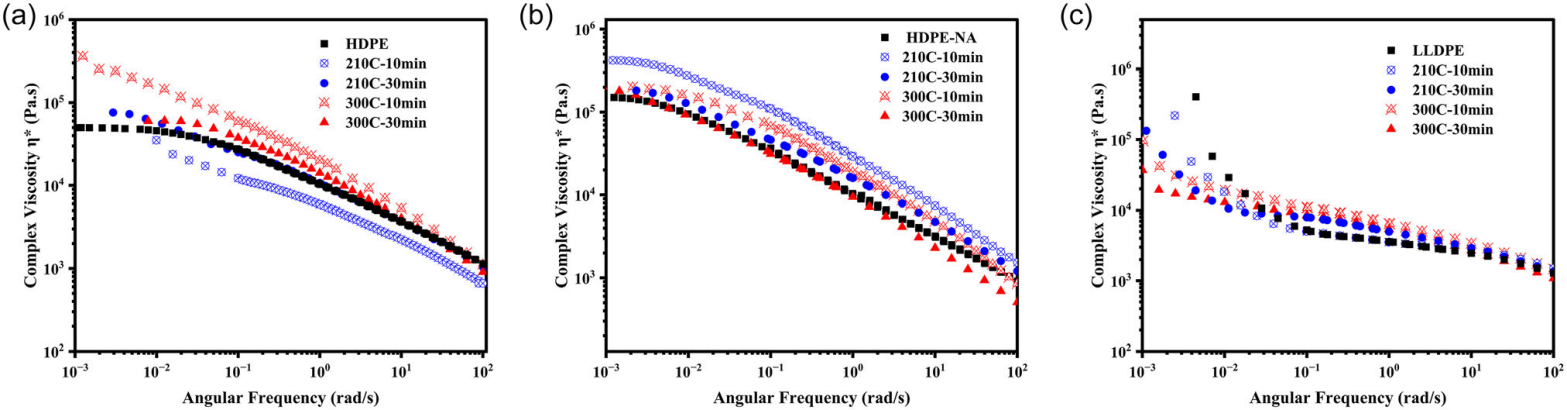
Complex viscosity after Brabender processing for (a) HDPE, (b) HDPE‐NA, and (c) LLDPE.

**FIGURE 3:**
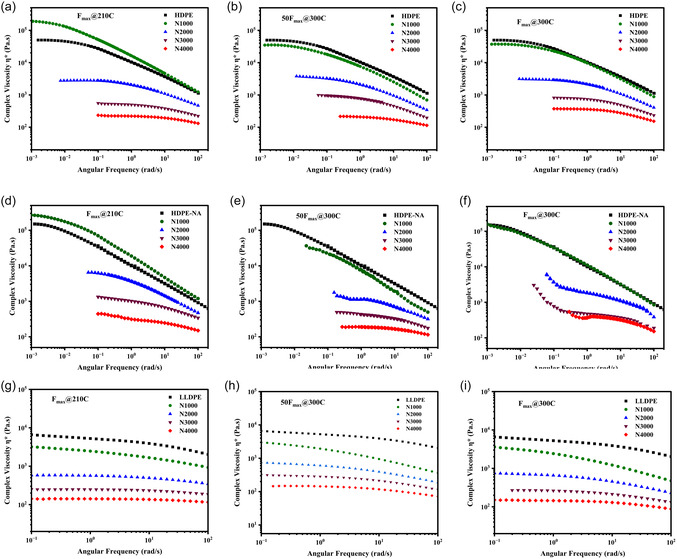
Complex viscosity after TSE processing under different processing parameters for (a–c): HDPE, (d–f): HDPE‐NA, (g–i): LLDPE.

The effect of processing parameters in the presence and absence of shear stress on evolving polyethylene structure was examined for two different melt processes, batch mixing and TSE, by measuring their complex viscosity, as shown in Figures 2 and 3. The complex viscosity changes only modestly with time and temperature alone in batch mixing (Figure 2a–c). For LLDPE, the upturn in the complex viscosity related to entanglement appears to lessen. It appears that thermal energy at low shear rates did not lead to a significant reduction in molecular weight for polyethylene, but it can be argued that it caused LLDPE's structure to change.

The temperature and time influenced structure evolution and viscosity much more significantly for the high shear rate TSE. Figure 3a–c illustrates the complex viscosity of HDPE, where a screw speed of 1000 rpm at a temperature of 210°C and maximum feeding rate actually increased the low–shear complex viscosity compared to the high processing temperature (N = 1000@300°C), suggesting a temperature‐induced chain growth or branching. At 300 C and for all higher screw speeds, the viscosity decreased compared to the reference material. Regardless of temperature, the viscosity shows similar values at the highest screw speed (N‐4000 rpm). This is likely due to viscous heating at the high screw speeds, bringing the temperatures higher than the nominal 210°C barrel temperature. Reducing the feeding rate to 50% of the maximum while keeping the processing temperature constant at 300°C did not significantly change the complex viscosity response. These results can be attributed to the prominent chain scission reactions that occur during high shear rate processing, outweighing the effect of temperature and high residence time (low feeding rate). Figure 3d–f shows the complex viscosity response of HDPE without additives (HDPE‐NA), generally similar to that of HDPE under processing conditions. However, slightly higher zero‐shear viscosities are observed for the F_max_@300C, likely due to the formation of more branched structures in the absence of additives compared to HDPE at the same processing condition. It should be noted that some oxidation could occur during the melt rheology measurements, contributing to the upturn at low frequency. However, since the measurements were conducted at 200°C and took less than 15 min, it is unlikely that this effect was significant, as indicated by a time sweep test provided in the supporting information (Figure S3). Figure 3g–i illustrates the viscosity of LLDPE after high shear rate processing at different temperatures and feeding rates (residence times). LLDPE exhibits less shear thinning across the tested frequency range due to its branched structure and narrower molecular weight distribution. However, it also displays the typical complex viscosity reduction with increasing screw speed. The dominant decrease in complex viscosity at the highest screw speeds implies that the mechanical energy promoted β‐scission reactions in polyethylene structure.

### 3.3 SEC

**FIGURE 4:**
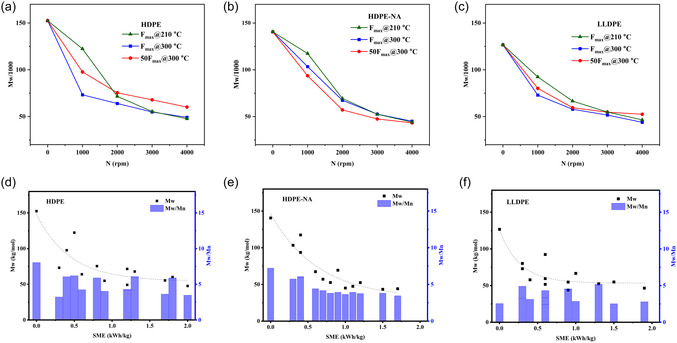
Molecular weight and polydispersity for PEs before and after TSE processing as a function of (a–c) processing parameters, and (d–f) SME.

The SEC result after a high shear TSE revealed general reduction in weight‐average molecular weight as the screw speed increased (Figure 4a–c). Interestingly, at higher screw speeds (higher shear rates), temperature did not significantly contribute to this reduction in all three materials. Although it was less obvious in the rheology results, SEC showed that the residence time increase (for 50F_max_) resulted in slightly more molecular weight reduction only in HDPE‐NA, highlighting the role of additives like stabilizers against degradation during melt processing. In both temperature and residence time assessments, the molecular weight reached similar levels at the highest screw speed. These results agree with the complex viscosity responses. The molecular weight and the polydispersity index (M_w_/M_*n*_) are shown as a function of SME in Figure 4d–f to highlight the combined influence of residence time and screw speed; it is evident that in HDPE and HDPE‐NA, the higher mechanical energy input preferentially shortened the high molecular weight portion of the distribution, leading to a narrower polydispersity. Full SEC distribution curves are provided in the supporting information (Figure S1).

### 3.4 FTIR

**FIGURE 5:**
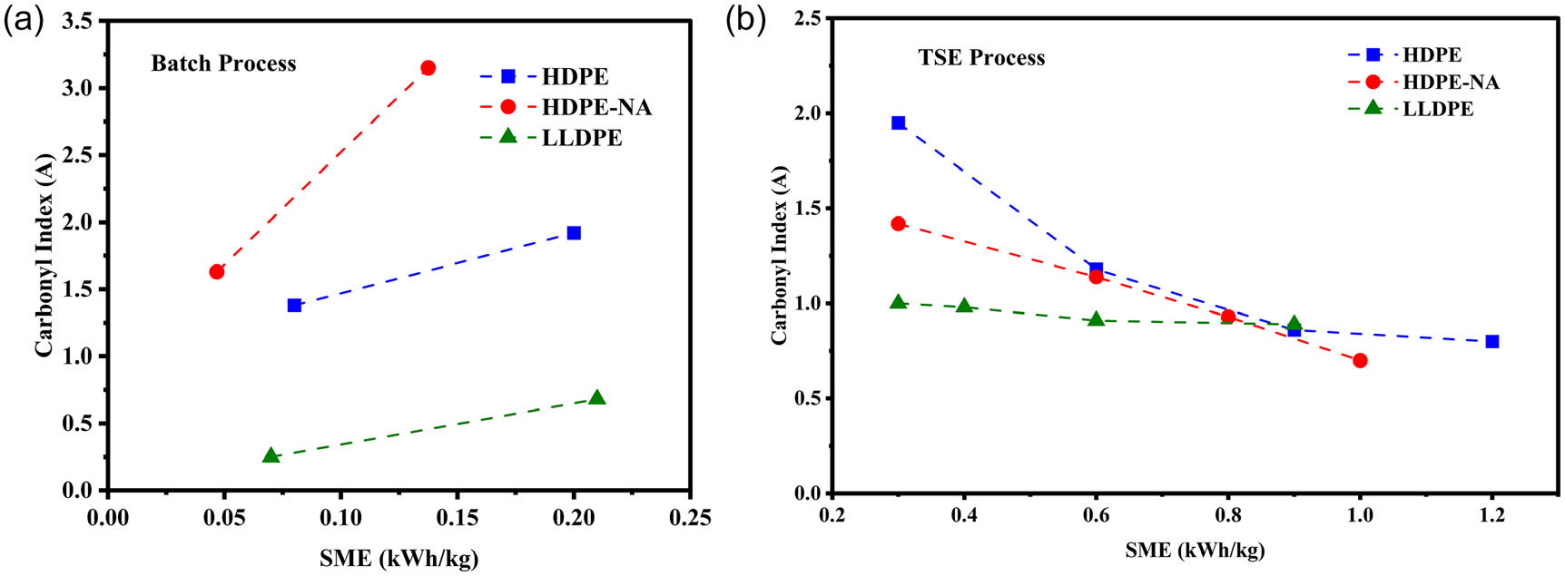
Carbonyl index intensity versus SME after (a) Batch @ 300°C and (b) TSE processing.

The chemical structure evolution was measured using FTIR spectroscopy, shown in Figure 5 (full spectra are provided in the supporting information, Figure S2). The spectra show the formation of new functional groups over time and temperature during batch mixing, as well as when subjected to high shear stress during the TSE process at 300°C and F_max_. Thermal‐oxidative reaction occurred, forming hydroxyl, carbonyl, ether, and ester functional groups as well as unsaturated carbon double bonds. While quantification of the degree of functionalization was not possible using the FTIR spectra, the intensity of these functional groups increased with time and temperature during the batch process. For the TSE, increasing screw speed and applying more shear stress did not result in more oxygen functionality formation. This is likely due to the greater exposure of polymer surface area to oxygen in the batch mixer compared to the closed barrel of the TSE. The intensity of the peak at 1740 cm^−1^ corresponding to the carbonyl functional group decreased, especially in HDPE‐NA grade, which is more prone to thermal oxidation due to absence of additives. This suggests that chain scission reactions were dominant over other chemical reactions in the high shear rate TSE process.

The correlation between the carbonyl index and SME during these two distinct processes is shown in Figure 5a,b. After Brabender processing at 300°C, the carbonyl index showed a direct correlation with SME, increasing across all polyethylene grades. Among them, HDPE‐NA exhibited the most significant thermal oxidation, while LLDPE demonstrated the lowest formation of carbonyl functional groups. In contrast, the carbonyl index decreased in TSE process with increasing SME. This finding confirms that mechanical energy input during the TSE process contributes to chain structure transformation in polyethylene, resulting in fewer thermal oxidative reactions due to the inherently lower residence time and oxygen exposure of this process. From TSE with much shorter residence times, it does not appear that the additives in the HDPE had any protection against formation of oxygen functionality, as the HDPE with additives was even more functionalized than the HDPE‐NA (especially at low SME). The reasoning for this is unclear and may require more study. It can also be speculated that the ester groups present in the phenolic antioxidants contributed to the total carbonyl index, though they are not evident in unprocessed material.

### 3.5 Catalytic Reactive Extrusion

**FIGURE 6:**
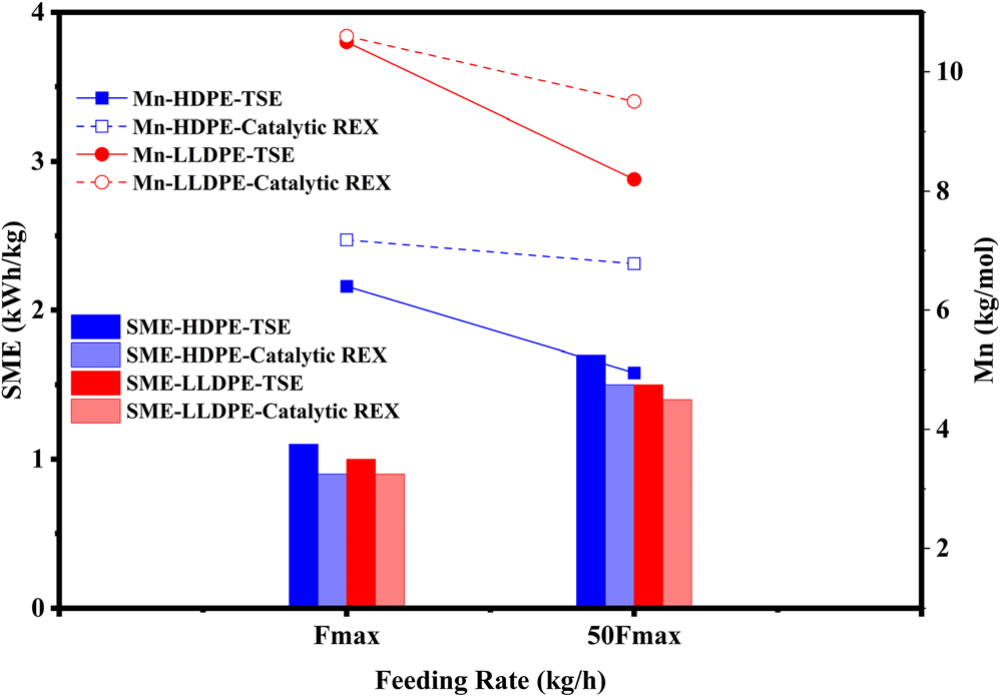
SME and Mn versus feeding rate in TSE and catalytic REX processes for HDPE and LLDPE.

The M*w* and M*n* reached a minimum value of approximately 50 and 15 kg/mol, respectively, across all grades after TSE processing. To achieve further molecular weight reduction, a reactive extrusion approach was employed by incorporating 10 wt% of zeolite‐containing catalyst E‐Cat under high shear stress conditions. Figure 6 shows that SME in both TSE and REX processes is mainly affected by residence time, with lower feeding rates increasing SME. When catalyst is added, the SME values are slightly lower, likely due to greater molecular weight and viscosity reduction. Increasing residence time also reduces Mn, similar to the trend observed for SME. However, the Mn values do not decrease as significantly in REX as when no catalyst is added, as shown by the less negative slopes for the open symbols in Figure 5. This suggests that the catalyst may act more specifically on chain ends, leaving most of the molecule intact. If the objective is to reduce the polymer molecular weight overall, perhaps mechanical stress alone should be conducted prior to addition of catalyst, at which point refinement of the product distribution could be accomplished. Further testing was conducted to clarify the distinctions between mechanical action and catalyst‐assisted processing.

### 3.6 Rheology

**FIGURE 7:**
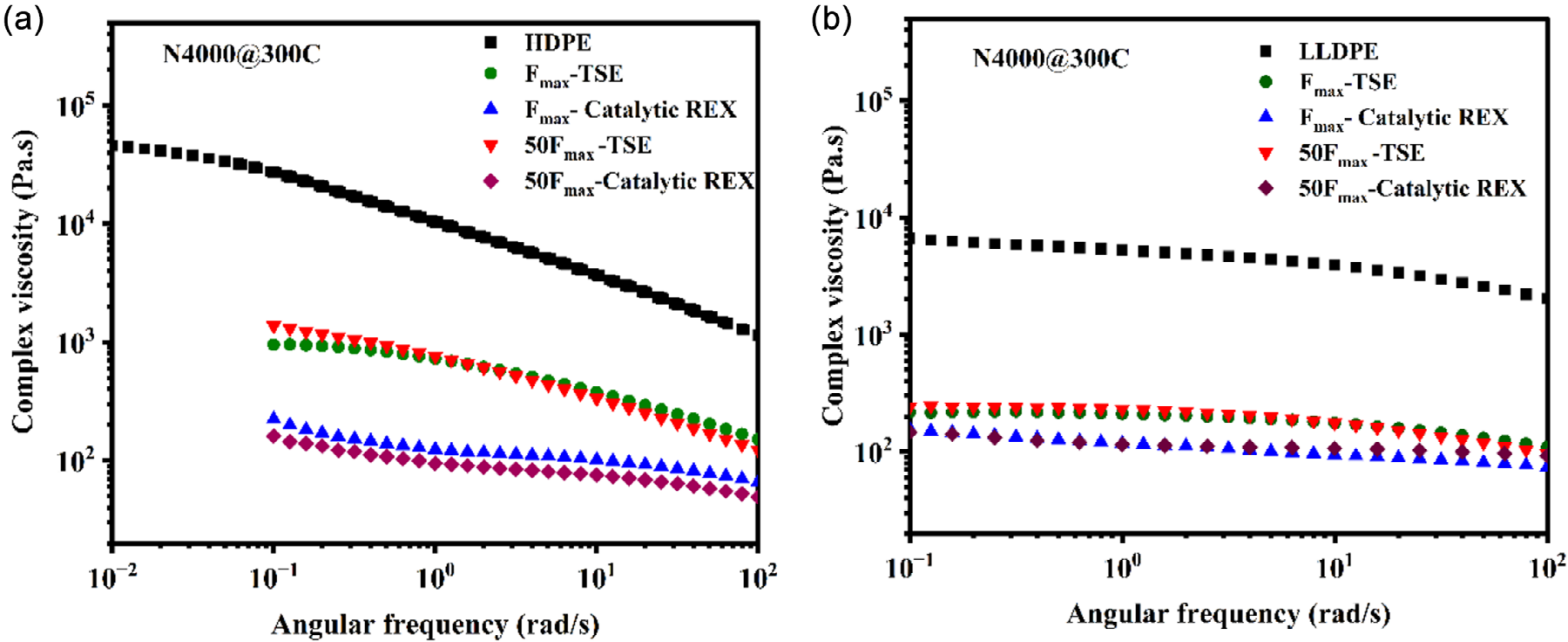
The complex viscosity after conducting the catalytic high‐shear reactive extrusion.

The complex viscosity of polyethylene after conducting the catalytic reactive extrusion process is illustrated in Figure 7a,b. The viscosity is continuing to decrease after adding the catalyst during the higher shear TSE process with a screw speed of 4000 rpm at 300°C. This result indicates intermolecular catalytic reactions occurred during extrusion processing, which leads to a reduction in molecular weight and consequently, a decrease in viscosity. Moreover, HDPE exhibits a higher change compared to LLDPE, and its viscosity is reduced more significantly under the same processing conditions.

### 3.7 FTIR

**FIGURE 8:**
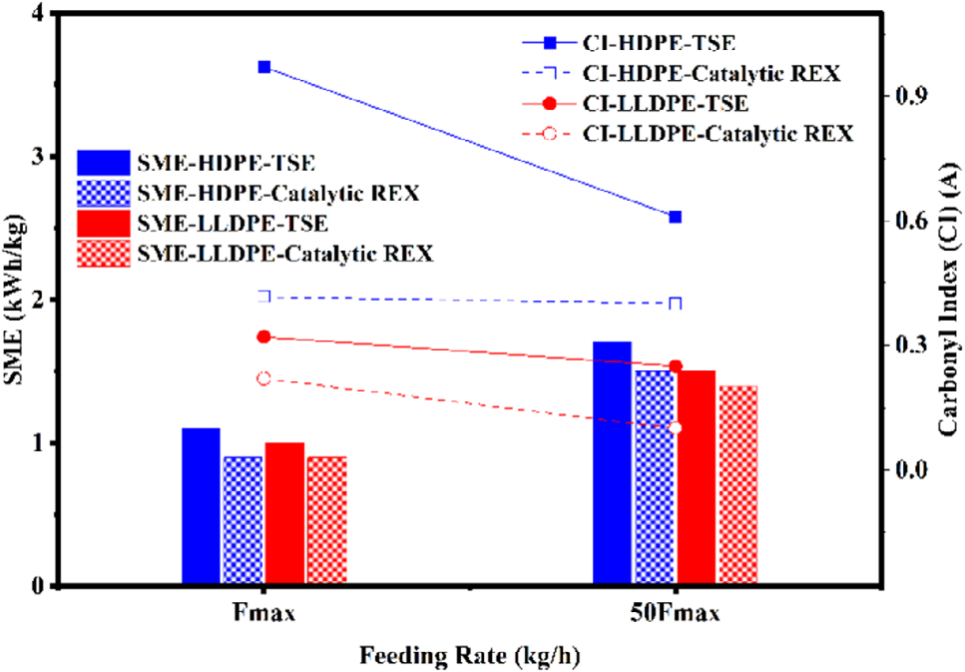
Chemical structure evolution of polyethylene after catalytic and high shear extrusion through comparison of carbonyl index with specific mechanical energy for all samples.

Polyethylene undergoes oxidative functionalization after the catalytic reactive extrusion processing, as illustrated in Figure 8 (full spectra are provided in the supporting information, Figure S4). Notably, the carbonyl functional groups, which appear at a wavenumber around 1740 cm^−1^, are more prominent at higher screw speeds. However, the peak intensity decreases after undergoing catalytic extrusion processing. This effect is particularly evident in HDPE grade, while there is no significant change in the carbonyl index for LLDPE grade, indicating its resistance to thermal oxidation reactions. On the other hand, the ether and ester functional groups, which appear at wavenumbers 1100 and 1200 cm^−1^, become more prominent after adding the catalyst in both grades. This suggests the catalyst has a contribution in oxidative reactions in addition to scission reactions, even if not major. Furthermore, the reduction in the carbonyl index observed under extended processing (longer residence time, 50%F_max_) and higher SME conditions again indicates that the intensive thermal‐mechanical environment promotes chain scission rather than oxygen incorporation.

### 3.8 ^13^C NMR

The chemical structural transformations of HDPE and LLDPE resulting from catalytic reactive extrusion were analyzed and compared for the TSE and batch processes using ^13^C NMR spectroscopy. The effect of the processing methodology is clearly distinguishable in the resultant structure, specifically concerning the length and frequency of chain branching and the distribution of saturated chain ends (CH_2_, CH_3_) and vinyl chain ends (RC = CH) and unsaturated fractions (trans RC = CR) within the polymer chains as outlined in Table 4. For HDPE, the REX process resulted in a significant increase in the formation of short‐chain branches (SCB), predominantly methyl branches, especially with the catalyst present. The HDPE showed more internal trans unsaturation compared to the noncatalytic REX process. Conversely, the noncatalytic REX predominantly generated long‐chain branches (LCB) and terminal vinyl chain‐end groups. These findings are consistent with a catalyst‐promoted mechanism: the high shear and acid catalyst facilitate β‐scission of the existing long branches and radical combination into double bonds and partial recombination or alkyl transfer reactions that generate the short, branched fragments [37]. For LLDPE, the primary influence of the catalyst during reactive TSE was on the further formation of the SCB. Notably, LLDPE did not exhibit the formation of LCB either with or without the catalyst during the TSE process, contrasting sharply with the behavior of HDPE. However, a common outcome for both PE grades was that the catalytic REX process reduced the concentration of terminal vinyl functional groups while simultaneously increasing the fraction of internal trans unsaturation, reinforcing the mechanism of catalyst‐driven double‐bond isomerization across both polyethylene types. The residence time with higher SME significantly progresses this chemical structure further in HDPE, while a minor effect is observed in LLDPE under TSE process with and without catalyst.

**TABLE 4:** Chemical structure fragments in HDPE and LLDPE from ^13^C NMR spectroscopy, normalized to 1000 total carbon atoms.

Material	Processing condition	Chemical groups				
		LCB*	SCB	Saturated chain end	Vinyl chain end	trans RC = CR
HDPE	HDPE	0.0	0.6	1.05	1.10	0.00
	F_max_‐TSE	1.6	0.7	3.23	1.15	0.42
	50F_max_‐TSE	2.5	0.6	4.51	1.15	0.55
	F_max_‐Catalytic REX	0.0	3.8	3.91	0.00	0.67
	50F_max_‐Catalytic REX	0.0	4.2	4.13	0.00	0.87
LLDPE	LLDPE	0.0	10.7	0.70	0.00	0.00
	F_max_‐TSE	0.0	10.3	1.90	0.78	0.20
	50F_max_‐TSE	0.0	10.9	2.52	0.88	0.29
	F_max_‐Catalytic REX	0.0	13.1	2.65	0.00	0.55
	50F_max_‐Catalytic REX	0.0	12.9	2.96	0.00	0.58

*Note:* *LCBs determined via ^13^C NMR may or may not be rheologically significant.

### 3.9 TGA

**FIGURE 9:**
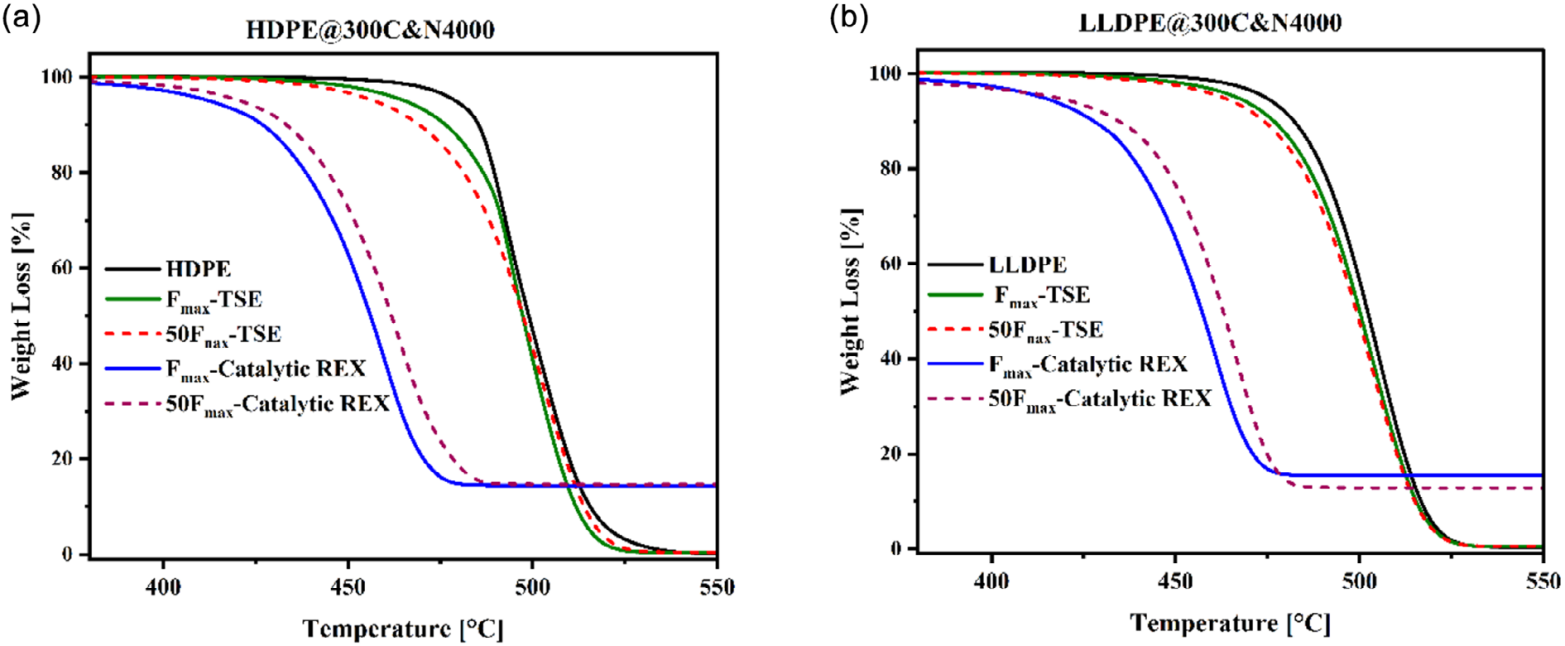
TGA result after catalytic reactive extrusion for (a) HDPE and (b) LLDPE.

Polyethylene's decomposition temperature decreased after catalytic reactive extrusion, as illustrated in Figure 9. After TSE processing, applying only high shear without the catalyst, the decomposition temperature of polyethylene is only slightly reduced. However, the decomposition onset temperature after catalytic reactive extrusion (where the polymer was in contact with the catalyst already for one heat cycle) is dramatically reduced. This reduction can be attributed to both residual activation of the catalyst and potentially the formation of weak bond functional groups like ethers and esters in the polyethylene backbone. The decomposition temperature of about 420°C suggests that the catalytic conversion of polyethylene could result in complete depolymerization at lower temperature.

### 3.10 HTGC

**FIGURE 10:**
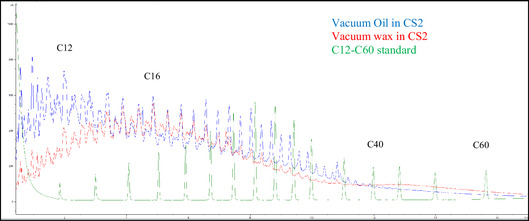
HT‐GC spectra of byproduct from catalytic reactive extrusion.

Hydrocarbon cracking analysis of the generated oil and wax from the vacuum trap and ducts from the devolatilization zone of the TSE revealed that the samples primarily consist of hydrocarbons in the C_12_–C_40_ range, with varying amounts of each compound. The oil fraction contains more compounds with fewer than 12 carbons, while the wax fraction contains more compounds with longer carbon chains, but mostly less than C_16_, as shown in Figure 10. This profile exhibits typical characteristics of polyethylene cracking.

## 4 Conclusion

This study found that catalytic reactive extrusion offers a highly efficient mechanochemical approach for depolymerizing and functionalizing polyethylenes, demonstrating the potential of high shear melt processing to address key limitations of conventional recycling methods. By consolidating processing conditions into the SME metric, we established that high‐intensity mechanical energy input is the primary driver of chain scission. Incorporating catalyst under these high‐shear conditions resulted in significant chemical restructuring in both HDPE and LLDPE, likely due to improved polymer‐catalyst contact and high localized temperature. Chemical analysis confirmed structural transformation in polyethylene, evident in the decreased thermal oxidative indicator (carbonyl index), the near‐elimination of terminal vinyl functional groups, and a simultaneous increase in internal unsaturation and short‐chain branches. While LLDPE exhibited greater inherent resistance to thermal oxidation, the catalytic process successfully altered the structure of both PE grades, generating volatile fractions of light oligomeric oils that represent valuable chemical intermediates.

Despite these promising results, several critical questions remain. Future work should explore the effect of varying catalyst loadings on reaction kinetics and product selectivity, as well as postprocess catalytic cracking to determine whether upstream mechanochemical activation can significantly lower thermal energy requirements for complete depolymerization. Additionally, achieving a comprehensive mass balance through improved collection and characterization of volatile byproducts is essential for evaluating process efficiency and environmental impact. Addressing these gaps will enable optimization of catalytic reactive extrusion as a preconditioning strategy for downstream chemical recycling, advancing its role as a solvent‐free, energy‐efficient pathway for circular polyolefin waste management.

## Supporting Information

Additional supporting information can be found online in the Supporting Information section.

## Funding

This study was supported by CPChem.

## Conflicts of Interest

The authors declare no conflicts of interest.

## Supporting information

Supplementary Material

## Data Availability

The data that support the findings of this study are available from the corresponding author upon reasonable request.
