# Supplementary material for: Catalytic High‐Speed Reactive Extrusion for Polyethylene Mechanochemical Upcycling
Source: ChemSusChem. 2026 Apr 26;19(8):e70622. doi: 10.1002/cssc.70622 (PMC13110911; doi:10.1002/cssc.70622)
Supplement: Supplementary file 1 — Supplementary Material [file CSSC-19-e70622-s001.pdf]

Supporting information

The screw profile for twin screw extruder was programmed as outlined in Table S1.

Table S1: Screw profile programming of twin-screw extruder. Elements are listed in order from feeding zone to die.

Type of screw element	Screw element detail		Number of elements
	Pitch (mm)	Length (mm)	
Forward flight	10	10	1
	25	25	7
	15	15	2
Kneading block	R/5	15	1
	N/5	15	1
	L/5	15	1
Reverse element	-15	7.5	1
Forward flight	25	25	3
	20	20	2
Kneading block	R/5	15	1
	N/5	15	1
	L/5	15	1
Forward flight	20	20	2
	20	10	1
	15	15	4
	10	15	1
	10	10	1
Kneading block	R/5	15	1
	N/5	15	1
	L/5	15	1
Forward flight	25	25	1
	20	20	1
Reverse element	-15	7.5	1
Forward flight	25	25	3
	20	20	4
	15	15	1
	10	5	2
	10	15	1

Molar mass distribution from SEC for HDPE, HDPE-NA, and LLDPE after TSE processing are presented in Figure S1.

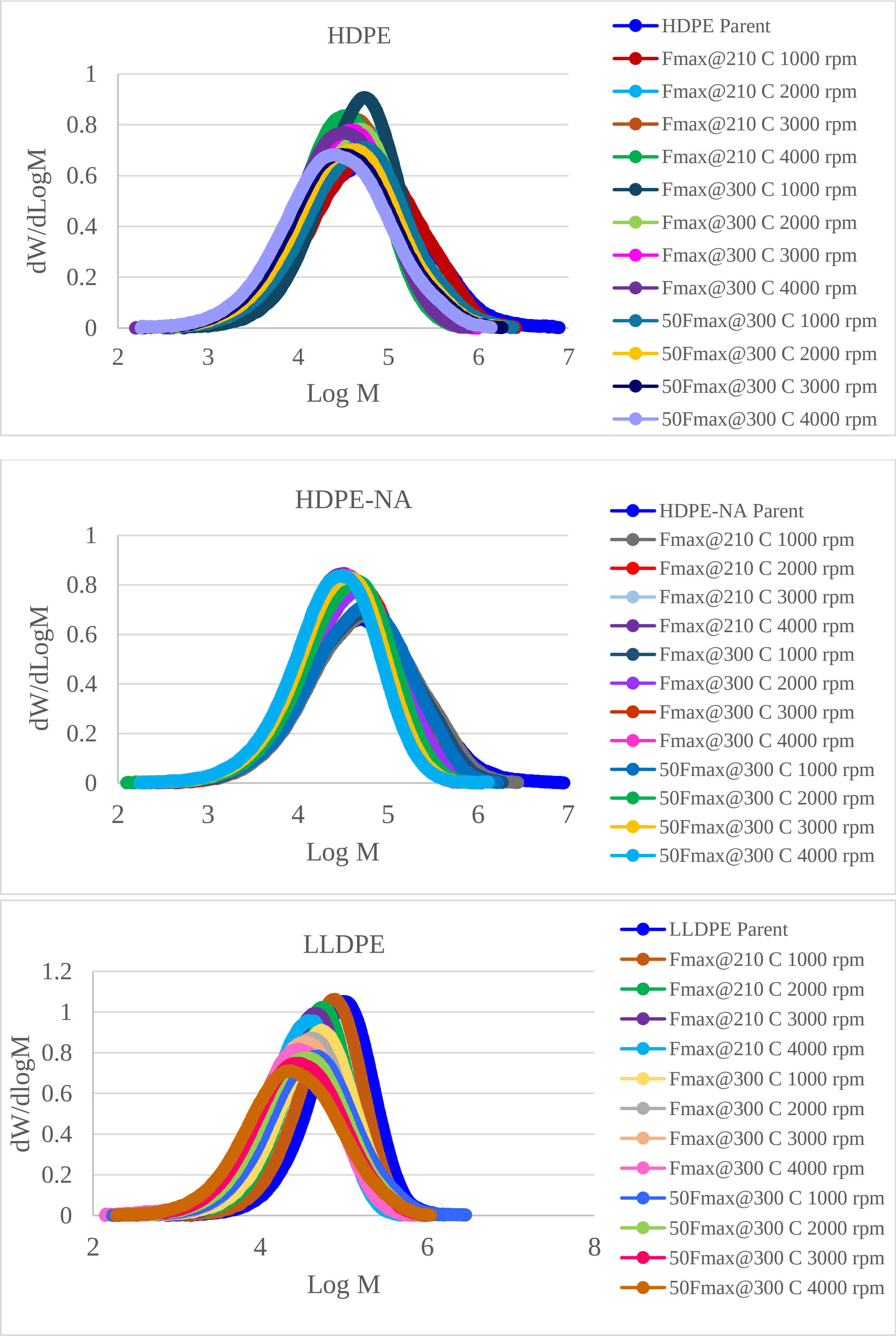

Figure S1: Molar mass distribution of all polyethylene after TSE processing

Figure S2 presents FTIR spectra of all polyethylene grades, illustrating the evolution of their chemical structure after Batch and TSE processing.

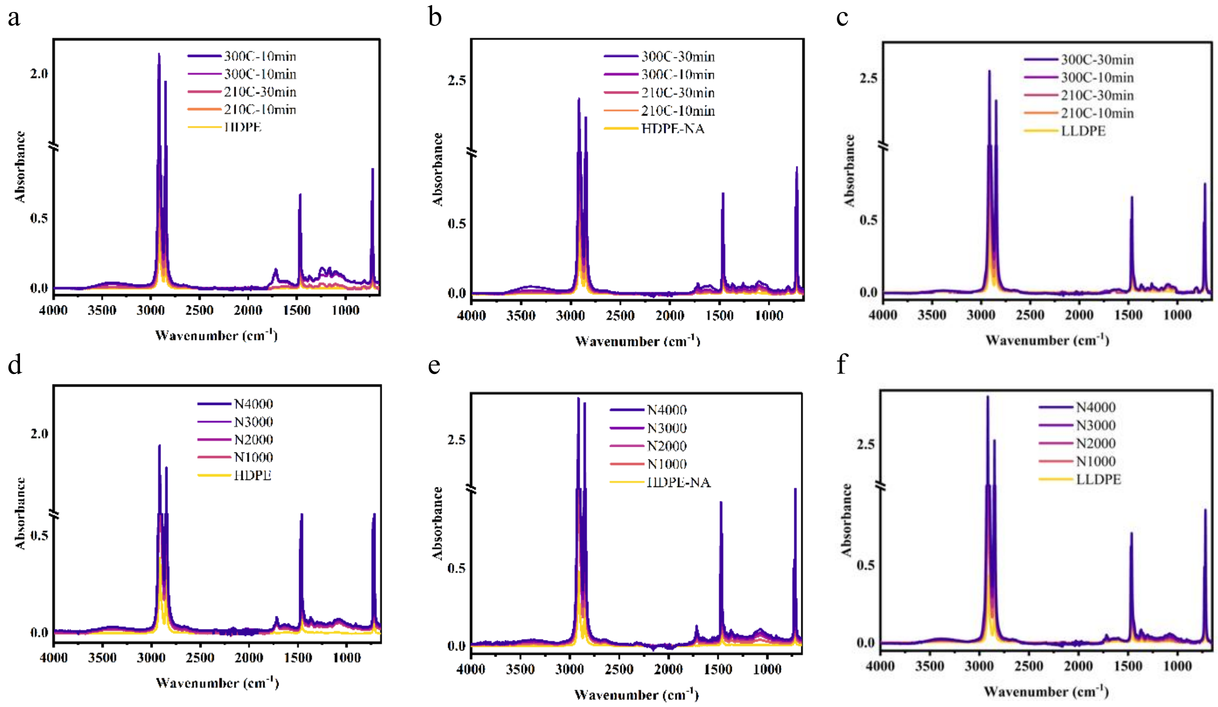

Figure S2: FTIR spectra of the three polyethylene variants after (a-c) Batch, and (d-f) TSE processing @ 300 °C.

The time sweep test for HDPE in nitrogen at 200°C, as shown in Figure S3, revealed a negligible change in its rheological behavior over the test duration, which is less than 15 minutes.

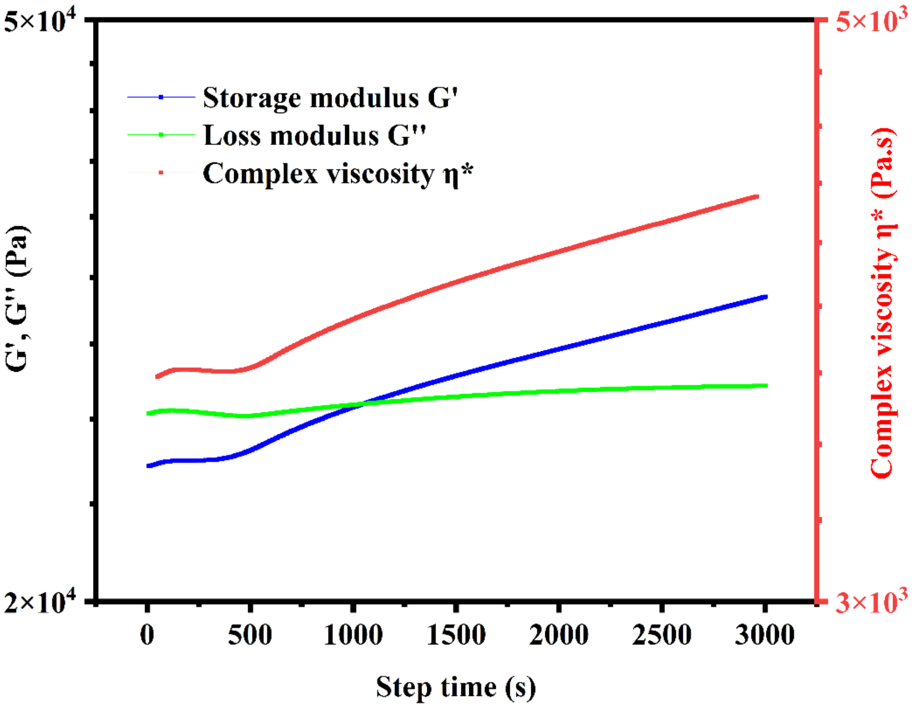

Figure S3: Time sweep test at 200 °C for HDPE.

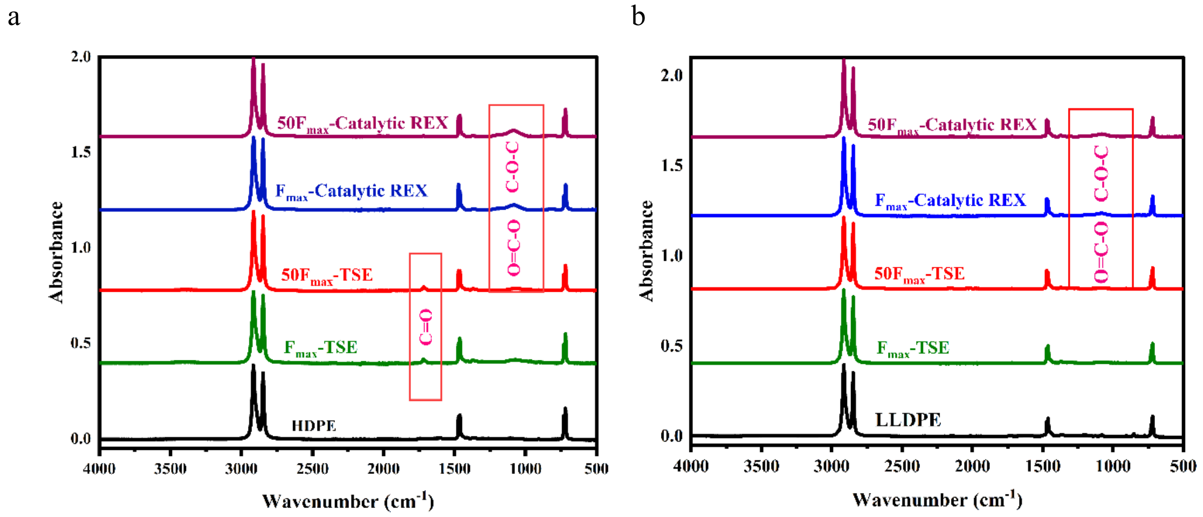

Figure S4: FTIR spectra indicating chemical structures evolution after catalytic and high shear extrusion for (a) HDPE, (b) LLDPE.

Figure S4 shows FTIR traces of HDPE and LLDPE after TSE and catalytic REX processing at various residence times.
